# Linear Algebraic Beamforming Design for Multiuser MISO Interference Channels: A Reduction in Search Space Dimension

**DOI:** 10.3390/e20060431

**Published:** 2018-06-04

**Authors:** Sang Won Choi, Won-Yong Shin, Juyeop Kim

**Affiliations:** 1 ICT Convergence Research Team, Korea Railroad Research Institute, Eiwang 16105, Korea; 2 Department of Computer Science and Engineering, Dankook University, Yongin 16890, Korea; 3 Department of Electronics Engineering, Sookmyung Women’s University, Seoul 04310, Korea

**Keywords:** beamformer design, correlated block fading, interference channel, linear algebraic beamforming, search space dimension

## Abstract

Near-optimal transmit beamformers are designed for multiuser multiple-input single-output interference channels with slowly time-varying block fading. The main contribution of this article is to provide a method for deriving closed-form solutions to effective beamforming in both low and high signal-to-noise ratio regimes. The proposed method basically leverages side information obtained from the channel correlation between adjacent coding blocks. More specifically, our methodology is based on a linear algebraic approach, which is more efficient than the optimal scheme based on the Gaussian input in the sense of reducing the average number of search space dimensions for designing the near-optimal transmit beamformers. The proposed method is shown to exhibit near-optimal performance via computer simulations in terms of the average sum-rate.

## 1. Introduction

### 1.1. Background

Today, people are living in the era of possessing a variety of small communications devices such as mobile phones, tablet computers, and wearable devices. Such devices are required to have the primary feature of miniaturization, which has still been taken into account as a fundamental issue to the most of manufacturers. Those devices, however, have practical challenges in the perspective of commercial implementation since they are allowed to deploy only a fewer number of antennas due to an inherent limitation of the physical size.

Nowadays, it is also a crucial requirement for several mobile devices to conduct communications and signal processing efficiently. People are in pervasive use of multiple devices with the support of high data rate and mobility. In this circumstance, it is inevitable to confront multiuser interference and to develop effective interference management and mitigation. It reveals that the radio resource such as time, frequency, and space needs to be well-shared for supporting multiple users in an efficient way.

For tackling the aforementioned two technical issues (i.e., physical limitation and interference), there have been intensive studies on investigating the fundamental limits of a multiuser multiple-input single-output (MISO) interference channel (IC) environment by designing achievable schemes [1,2,3,4,5,6,7,8,9]. Recently, the optimality of transmit beamforming under the assumption of single-user detection has been independently studied in [3,4,5]. In addition, efficient beamforming designs have been studied in terms of reducing the search space dimension along with either closed forms (e.g., [3,4,5,8,9]) or numerical algorithms (e.g., [2,3,6,7,9]).

From the above studies, insightful observations can be made as follows:

- Transmit beamforming is Pareto-optimal [10,11,12], which can be represented as a weighted linear sum of maximal ratio combining (MRC) and zero-forcing (ZF) beamformers [1].
- The closed-form solution to transmit beamforming is feasible, which is attained based on the approach to converting the non-convex optimization problem into several convex optimization problems [3,4,5].
- Transmit covariance matrices with rank 1 are sufficient to achieve all points on the Pareto boundary under the assumption of single-user detection with the Gaussian input [3,4,5].
- According to the uplink-downlink duality [13] beyond the framework transformed by convex optimization, the number of optimized parameters can be further reduced in searching for the optimal transmit beamforming vectors [8].

On the other hand, there have been a lot of research on the MISO ICs for practical use [14,15,16,17,18]. Specifically, beamforming design [14,15,16] and power allocation [17,18] issues have been studied for enhancing outage performance under an imperfect CSI. Please note that the transmit strategy and power allocation are main factors for maximizing weighted sum-rate or outage rate under perfect CSI and imperfect CSI, respectively. In this paper, we focus on the practical beamforming design for the multiuser MISO IC with time-varying fading assuming the perfect CSI, which gives an insight on whether linear algebraic approach for the proposed beaforming design is feasible or not.

### 1.2. Main Contributions

In this article, we aim at designing near-optimal transmit beamformers for a multiuser MISO IC with slowly time-varying block fading (A part of this paper was presented at the ICTC in 2014 [19]). We provide a method for deriving a closed form solution to effective beamforming. While the essential idea behind the prior approaches to designing the optimal transmit beamformers is to utilize each of channel instances in a separate manner, the proposed method basically leverages side information by *jointly* considering the channel correlation between adjacent coding blocks. More specifically, our methodology is based on a linear algebraic approach, which is more efficient than the optimal scheme based on the Gaussian input in the sense of reducing the average number of search space dimensions for designing the near-optimal transmit beamformers. As a main result, closed-form solutions to effective beamforming are provided in both low and high signal-to-noise ratio (SNR) regimes. We show that the proposed method exhibits near-optimal performance via computer simulations in terms of the average sum-rate. Note that for two-user multiple-input multiple-output (MIMO) ICs, beamforming update algorithms have been proposed using a convex approach. However, the algorithms are limited in practice since they cannot be applied to an arbitrary number of users [20].

### 1.3. Organization

The rest of this paper is organized as follows. Section 2 describes the system model. The proposed near-optimal transmit beamforming method is presented in Section 3. Its computational complexity is shown in Section 4. Numerical evaluation is shown via computer simulations in Section 5. Finally, we conclude the paper in Section 6.

For notational convenience, we use the following notations.

- Boldface letters x and X represent vector and matrix, respectively.
- $0_{M\times N}$ is M×N zero matrix.
- We use the user index *k*, where k∈{1,2,⋯,K}.
- The transmit signal vector is denoted as $x^{\left[k\right]}\left(t\right)\in C^{M}$ of the *k*th user for the *t*th coding block.
- The channel vector from the *j*th transmitter to the *k*th receiver is denoted as $h^{\left[kj\right]}\left(t\right)\in C^{1\times M}$, where the *i*th component $h_{i}^{\left[kj\right]}\left(t\right)$ of $h^{\left[kj\right]}\left(t\right)$ is the channel coefficient from the *i*th antenna at the *j*th transmitter to the *k*th receiver.
- The noise vector is denoted as $n^{\left[k\right]}\left(t\right)\in C$ for the *t*th coding block.

## 2. System Model

We consider a multiuser MISO IC with slowly-varying block fading, where *M* antennas are deployed at each transmitter and a single antenna is used at each receiver. Each coding block experiences correlated block fading across a time domain while the channel coefficient is fixed within a block. Typically, the correlated fading channel comes from the Doppler effect due to a mobile environment [21]. The channel correlation value is usually estimated from a sample average in the time domain, where the sample is given by a multiplication of two adjacent channel coefficients normalized by a multiplication of the two channel coefficients’ magnitudes [22]. Then, the received signal of the *k*th user for the *K*-user MISO fading IC for the *t*th coding block is expressed as

(1) $$\begin{array}{c} y^{\left[k\right]}\left(t\right)=h^{[kk]†}\left(t\right)x^{\left[k\right]}\left(t\right)+\sum_{j=1,j\neq k}^{K}h^{[kj]†}\left(t\right)x^{\left[j\right]}\left(t\right)+n^{\left[k\right]}\left(t\right) \end{array}$$

where $x^{\left[k\right]}\left(t\right)\in C^{M}$ is assumed to be circularly symmetric complex Gaussian with zero-mean and covariance $S^{\left[k\right]}\left(t\right)$. Also, we assume that any two channel vector pairs from all channel vectors $h^{\left[kj\right]}\left(t\right)$s for ∀k,j∈{1,⋯,K} are linearly independent of each other, which is valid when all channel vectors $h^{\left[kj\right]}$s are independent and identically distributed (i.i.d.) from a continuous distribution. In addition, two channel vectors $h^{\left[kj\right]}\left(t-1\right)$ and $h^{\left[kj\right]}\left(t\right)$ for adjacent coding blocks are assumed to be highly correlated with each other. The noise $n^{\left[k\right]}\left(t\right)\in C$ is circularly symmetric complex Gaussian with zero mean and unit variance. We basically assume that perfect global CSI is available at the transmitters so that each transmitter acquires $h^{\left[kj\right]}\left(t\right)$s for ∀k,j∈{1,⋯,K}. We also assume that receivers have perfect local CSI so that the *k*th receiver acquires $h^{\left[kj\right]}\left(t\right)$s for ∀j∈{1,⋯,K}.

## 3. The Design of Proposed Near-Optimal Transmit Beamformers

In this section, we describe the design methodology of our proposed transmit beamformers. Our method is built upon the idea of periodic updates [5] in transmit beamforming. To design near-optimal periodically updated transmit beamformers, the proposed method basically leverages the effective side information, which corresponds to the received signal-to-interference-plus-noise ratio (SINR) for the previous coding block.

More specifically, our main idea is to design transmit beamforming vectors for the present coding block using the optimal ones found at the previous coding block. Under the assumption that two channel vectors for adjacent coding blocks are highly correlated, the received SINR for the previous coding block can be approximately the same as that for the present coding block. That is, the SINR at the *j*th receiver for the *t*th coding block can be approximated as

(2) $$\begin{array}{c} \frac{{\left|h^{[jj]†}\left(t-1\right)v^{\left[j\right]}\left(t-1\right)\right|}^{2}P^{\left[j\right]}}{1+\sum_{k=1,\;k\neq j}^{K}{\left|h^{[jk]†}\left(t-1\right)v^{\left[k\right]}\left(t-1\right)\right|}^{2}P^{\left[k\right]}}\\ ≃\frac{{\left|h^{[jj]†}\left(t\right)\left(\frac{v^{\left[j\right]}\left(t-1\right)+\Delta_{v^{\left[j\right]}\left(t\right)}}{∥v^{\left[j\right]}\left(t-1\right)+\Delta_{v^{\left[j\right]}\left(t\right)}∥}\right)\right|}^{2}P^{\left[j\right]}}{1+\sum_{k=1,\;k\neq j}^{K}{\left|h^{[jk]†}\left(t\right)\left(\frac{v^{\left[k\right]}\left(t-1\right)+\Delta_{v^{\left[k\right]}\left(t\right)}}{∥v^{\left[k\right]}\left(t-1\right)+\Delta_{v^{\left[k\right]}\left(t\right)}∥}\right)\right|}^{2}P^{\left[k\right]}} \end{array}$$

for ∀j∈{1,⋯,K}. Here, $v^{\left[k\right]}\left(t-1\right)$ is the beamforming vector at the *k*th transmitter for the (t−1)th coding block, $\Delta_{v^{\left[k\right]}\left(t\right)}$ is a change in the beamforming vector during two adjacent coding blocks (i.e., the (t−1)th and *t*th coding blocks), and $P^{\left[k\right]}$ is the transmit power at the *k*th transmitter, where ${∥v^{\left[k\right]}\left(t-1\right)∥}^{2}=1$. Please note that the transmit beamforming vector at the *k*th transmitter for the present coding block is given by

(3) $$\begin{array}{c} \frac{v^{\left[k\right]}\left(t-1\right)+\Delta_{v^{\left[k\right]}\left(t\right)}}{∥v^{\left[k\right]}\left(t-1\right)+\Delta_{v^{\left[k\right]}\left(t\right)}∥}, \end{array}$$

where $\Delta_{v^{\left[k\right]}\left(t\right)}\to 0_{M\times 1}$ and $∥v^{\left[k\right]}\left(t-1\right)+\Delta_{v^{\left[k\right]}\left(t\right)}∥\to 1$ as $\Delta_{h^{\left[kj\right]}\left(t\right)}\to 0_{M\times 1}$. Here, $0_{x\times 1}$ denotes the zero vector whose size is *x* and $\Delta_{h^{\left[kj\right]}\left(t\right)}$ denotes a change in the channel vector during two adjacent coding blocks.

Due to an ease of implementation, the interference at the receivers is treated as noise, which is a widely-adopted assumption in the literature. We employ single- user detection with Gaussian codebook constraints using separate coding. In the following two subsections, we focus on a linear algebraic approach for finding near-optimal transmit beamformers in low and high SNR regimes, instead of directly solving the original problem with highly correlated block fading.

### 3.1. Low SNR Approximation

In the low SNR regime, noise becomes more dominant than multiuser interference. Consequently, we have the following approximation from (2):

(4) $$\begin{array}{c} h^{[kk]†}\left(t-1\right)v^{\left[k\right]}\left(t-1\right)≃h^{[kk]†}\left(t\right)\left(v^{\left[k\right]}\left(t-1\right)+\Delta_{v^{\left[k\right]}\left(t\right)}\right),\;\forall k\in \left\{1,\;2,\;\cdots ,\;K\right\}. \end{array}$$

After some manipulations, Equation (4) can be expressed as the following matrix form:

(5) $$\begin{array}{c} A\cdot \Delta_{v}≃b, \end{array}$$

where

(6) $$\begin{array}{c} A=\left(\begin{array}{ccccc} h^{[11]†}\left(t\right) & 0_{1\times M} & \cdots & \cdots & 0_{1\times M}\\ 0_{1\times M} & h^{[22]†}\left(t\right) & 0_{1\times M} & \cdots & 0_{1\times M}\\ \vdots & 0_{1\times M} & \ddots & \cdots & \vdots \\ \vdots & \vdots & \vdots & \ddots & \vdots \\ 0_{1\times M} & 0_{1\times M} & \cdots & \cdots & h^{[K,K]†}\left(t\right) \end{array}\right), \end{array}$$

(7) $$\begin{array}{c} \;\Delta_{v}=\left(\begin{array}{c} \Delta_{v^{\left[1\right]}\left(t\right)}\\ \Delta_{v^{\left[2\right]}\left(t\right)}\\ \vdots \\ \Delta_{v^{\left[K\right]}\left(t\right)} \end{array}\right), \end{array}$$

and

(8) $$\begin{array}{c} b=\left(\begin{array}{c} h^{[11]†}\left(t-1\right)v^{\left[1\right]}\left(t-1\right)-h^{[11]†}\left(t\right)v^{\left[1\right]}\left(t-1\right)\\ h^{[22]†}\left(t-1\right)v^{\left[2\right]}\left(t-1\right)-h^{[22]†}\left(t\right)v^{\left[2\right]}\left(t-1\right)\\ \vdots \\ h^{[K,K]†}\left(t-1\right)v^{\left[K\right]}\left(t-1\right)-h^{[K,K]†}\left(t\right)v^{\left[K\right]}\left(t-1\right) \end{array}\right). \end{array}$$

Here, the sizes of matrices A, $\Delta_{v}$, and b are K×KM, KM×1, and K×1, respectively. Thus, in the low SNR regime, Equation (5) is an under-determined equation, which results in the following approximated least squares solution:

(9) $$\begin{array}{c} \Delta_{v}=A^{†}{\left(AA^{†}\right)}^{-1}b. \end{array}$$

### 3.2. High SNR Approximation

In the high SNR regime, the noise variance becomes negligible in comparison with the multiuser interference power. Consequently, Equation (2) can be approximated as

(10) $$\begin{array}{c} \frac{h^{[jk]†}\left(t-1\right)v^{\left[k\right]}\left(t-1\right)}{h^{[jj]†}\left(t-1\right)v^{\left[j\right]}\left(t-1\right)}≃\frac{h^{[jk]†}\left(t\right)\left(v^{\left[k\right]}\left(t-1\right)+\Delta_{v^{\left[k\right]}\left(t\right)}\right)}{h^{[jj]†}\left(t\right)\left(v^{\left[j\right]}\left(t-1\right)+\Delta_{v^{\left[j\right]}\left(t\right)}\right)} \end{array}$$

for ∀k≠j∈{1,⋯,K}. For notational convenience, we let

$$g^{\left[i,j,k,l,m\right]}\left(t\right)≜h^{[ij]†}\left(t-1\right)v^{\left[k\right]}\left(t-1\right)h^{[l,m]†}\left(t\right)$$

and

$$f^{\left[i,j,k,l,m,n\right]}\left(t\right)≜h^{[ij]†}\left(t-1\right)v^{\left[k\right]}\left(t-1\right)h^{[lm]†}\left(t\right)v^{\left[n\right]}\left(t-1\right).$$

Then, similar to Equation (5), Equation (10) can be rewritten as the following matrix form:

(11) $$\begin{array}{c} C\cdot \Delta_{v}=d, \end{array}$$

where

(12) $$\begin{array}{c} C=\left(\begin{array}{cccc} g^{\left[1,2,2,1,1\right]}\left(t\right) & -g^{\left[1,1,1,1,2\right]}\left(t\right) & \cdots & 0_{1\times M}\\ \vdots & \vdots & \ddots & \vdots \\ g^{\left[1,K,K,1,1\right]}\left(t\right) & 0_{1\times M} & \cdots & -g^{\left[1,1,1,1,K\right]}\left(t\right)\\ \vdots & \vdots & \vdots & \vdots \\ -g^{\left[K,K,K,K,1\right]}\left(t\right) & 0_{1\times M} & \cdots & g^{\left[K,1,1,K,K\right]}\left(t\right)\\ 0_{1\times M} & -g^{\left[K,K,K,K,2\right]}\left(t\right) & \cdots & g^{\left[K,2,2,K,K\right]}\left(t\right)\\ \vdots & \vdots & \ddots & \vdots \\ 0_{1\times M} & 0_{1\times M} & \cdots & g^{\left[K,K-1,K-1,K,K\right]}\left(t\right) \end{array}\right) \end{array}$$

and

(13) $$\begin{array}{c} d=\left(\begin{array}{c} f^{\left[1,1,1,1,2,2\right]}\left(t\right)-f^{\left[1,2,2,1,1,1\right]}\left(t\right)\\ \vdots \\ f^{\left[1,1,1,1,K,K\right]}\left(t\right)-f^{\left[1,K,K,1,1,1\right]}\left(t\right)\\ \vdots \\ f^{\left[K,K,K,K,1,1\right]}\left(t\right)-f^{\left[K,1,1,K,K,K\right]}\left(t\right)\\ \vdots \\ f^{\left[K,K,K,K,K-1,K-1\right]}\left(t\right)-f^{\left[K,K-1,K-1,K,K,K\right]}\left(t\right) \end{array}\right). \end{array}$$

Here, the sizes of C, $\Delta_{v}$, and d are K(K−1)×KM, KM×1, and K(K−1)×1, respectively. Thus, in the high SNR regime, Equation (11) becomes an overdetermined (underdetermined) linear equation [23] when (K−1)≥M ((K−1)<M), which results in the following approximated least squares solution:

(14) $$\begin{array}{c} \Delta_{v}=C^{†}{\left(CC^{†}\right)}^{-1}d. \end{array}$$

## 4. Computational Complexity

In this section, we show the computational complexity of the proposed beamformer and the conventional beamformers in [1,5,8] in terms of search space dimension. For the *K*-user MISO IC, one of the most dominant factors in computational complexity for finding the conventional beamformers is a search space dimension, which is associated with the number of optimized variables. This is because the conventional beamformers inevitably need to accomplish exhaustive search over variables to be optimized. Please note that the dominance of the search space dimension becomes significant for large *K*.

The conventional beamformers in [1,5,8] have focused on reducing the search space dimension for each channel instance separately. However, when additional side information is available under correlated channel environments, it is possible to reduce the number of optimized variables in the design of transmit beamformers. Specifically, instead of conducting optimization at each channel instance, the proposed method leverages the received SINR for the previous coding block as side information. Based on such information, we are capable of finding near-optimal transmit beamformers, which correspond to approximated least squares solutions. Consequently, it is observed from Equations (9) and (14) that the number of optimized variables (i.e., the search space dimension) is zero when we assume that the side information is available.

For the considered *K*-user MIMO IC, it may be necessary to update optimal transmit beamformers periodically. However, without the periodic update of the transmit beamformers, the search space dimension of the proposed beamformers is zero. Table 1 summarizes the search space dimension of the proposed beamformer in comparison with the conventional beamformers in [1,5,8], where the state-of-the-art method in [8] has the search space dimension of 2K−2 by optimizing 2K−2 real-valued parameters. Please note that the period of updating transmit beamformers can be expanded without substantial performance degradation, which will be numerically verified in the next section. The update period can be chosen from an acceptable level of channel correlation, which is typically calculated using the sample mean of correlation between two adjacent channel vectors in practice.

## 5. Numerical Evaluation

In this section, we show numerical results to validate that the proposed beamformers lead to satisfactory performance on the sum-rates with a remarkable reduction in search space dimension in comparison with the conventional beamformers.

### 5.1. Simulation Environments

Under the assumption of single-user detection with Gaussian codebook constraints, we present two benchmark schemes for comparison. First, “Optimal scheme” corresponds to the optimal transmit beamforming solution found at each channel instance and is obtained by maximizing the sum-rate using the SINR metric. Thus, “Optimal scheme” is also referred to as “MAX-SINR with adaptation”. In addition, we show the performance of “MAX-SINR with noadaptation” that does not perform updates of a transmit beamformer.

To model our correlated block fading in simulations, we adopt the autoregressive (AR) model [23] of order 1. Even if performance of the proposed scheme can be evaluated along with any correlated block channel models, we use the simplified AR model to effectively get an insight on the channel correlation. Then, the channel vectors $h^{\left[kj\right]}\left(t-1\right)$ and $h^{\left[kj\right]}\left(t\right)$ for two adjacent coding blocks satisfy the following relationship:

(15) $$\begin{array}{ccc} h^{\left[kj\right]}\left(t\right) & = & h^{\left[kj\right]}\left(t-1\right)+\Delta_{h^{\left[kj\right]}\left(t\right)}\\ & = & \rho h^{\left[kj\right]}\left(t-1\right)+\sqrt{1-\rho^{2}}u^{\left[kj\right]}\left(t\right), \end{array}$$

where $\Delta_{h^{\left[kj\right]}\left(t\right)}$ is a change in the channel vector during two adjacent coding blocks, ρ represents the correlation coefficient between adjacent channel vectors (i.e., $h^{\left[kj\right]}\left(t-1\right)$ and $h^{\left[kj\right]}\left(t\right)$), and each element of $u^{\left[kj\right]}\left(t\right)$s is i.i.d. and is circularly symmetric complex Gaussian with zero mean and unit variance. We generate channel vectors $h^{\left[kj\right]}\left(t\right)$ according to Equation (15), where $h^{\left[kj\right]}\left(t\right)$ is circularly symmetric complex Gaussian distributed with zero mean and covariance $I_{M\times M}$. It is assumed that absolute values of all the elements of the channel vectors are bounded between a nonzero minimum value and a finite maximum value.

Now, we address our cell deployment scenarios for effectively managing interference. We assume that cells are deployed such that each receiver experiences one dominant interfering link. For each desired link and one dominant interfering link, we apply the AR model in Equation (15) under the slow varying and small scale fading. In this setup, we evaluate the average sum-rates of the proposed scheme in comparison with the above two benchmark schemes.

**Figure 1:**
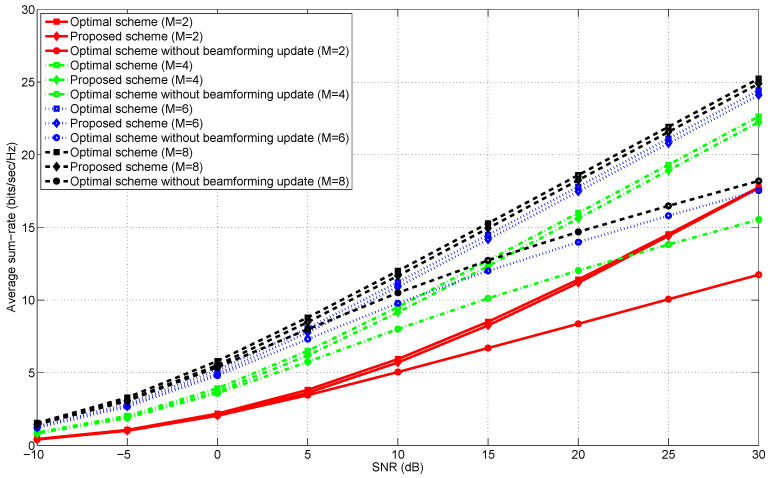
The average sum-rates versus SNR in the 2-user MISO IC for M∈{2,4,6,8} and ρ=0.9.

**Figure 2:**
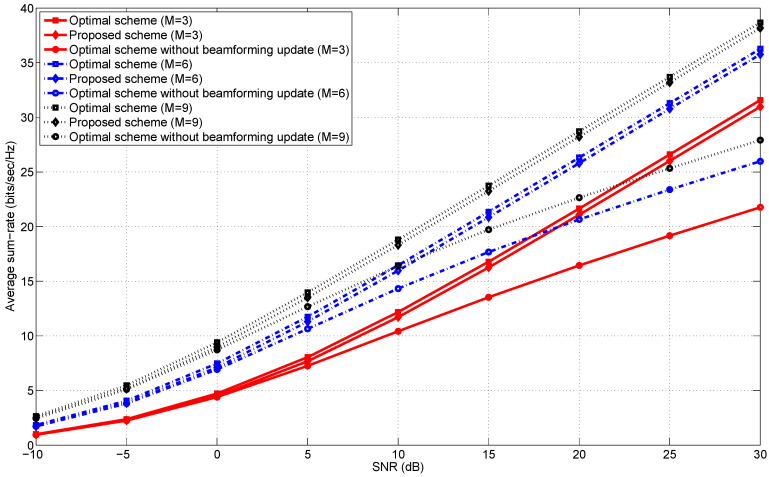
The average sum-rates versus SNR in the 3-user MISO IC for M∈{3,6,9} and ρ=0.9.

**Figure 3:**
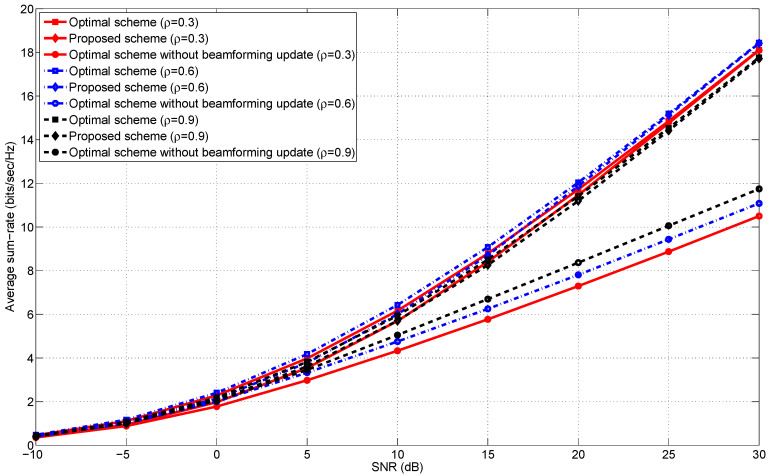
The average sum-rates versus SNR in the 2-user MISO IC for ρ∈{0.3,0.6,0.9} and M=2.

**Figure 4:**
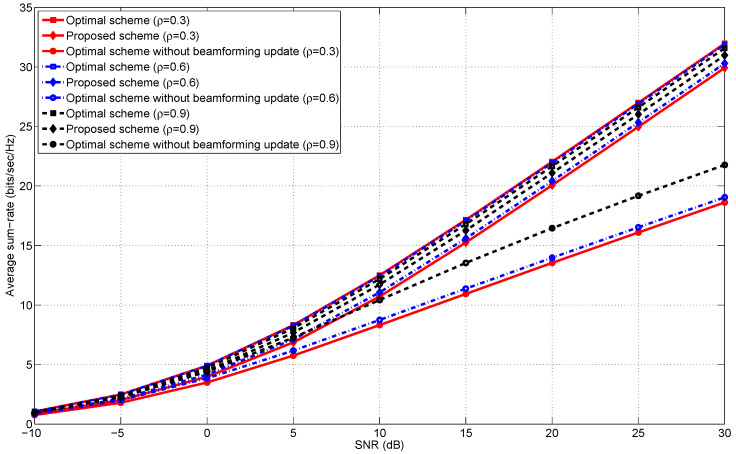
The average sum-rates versus SNR in the 3-user MISO IC for ρ∈{0.3,0.6,0.9} and M=3.

Figure 1, Figure 2, Figure 3 and Figure 4 illustrate the average sum-rates according to SNR for various *M*s (the number of transmit antennas) and ρs (the correlation coefficient). We closely investigate how the average sum-rates behave according to the following arguments.

- Low and high SNR regimes.
- The number of transmit antennas.
- The correlation coefficient ρ in Equation (15).

### 5.2. Low and High SNR Regimes

In an information-theoretic perspective [24,25], when M≥K (e.g., M≥3 with K=3), the optimal multiplexing gain [26] of *K* is achievable using the MAX-SINR scheme. Thus, we get a finite array gain (or equivalently, a power gain) by increasing the number of transmit antennas as shown in Figure 1 and Figure 2. Even though the average sum-rate of “MAX-SINR with no adaptation” also increases with respect to *M* and SNR, an increasing rate with respect to SNR (i.e., a slope of each curve in the high SNR regime) is lower than that of “Optimal scheme” and “Proposed scheme” since there is no update of transmit beamformers.

It is worth noting that the proposed scheme exhibits the near-optimal sum-rate performance for all SNR regimes and various *M*s. From this observation, we may conclude that the SINRs at all the receivers are expected to be sufficient side information for achieving near-optimal transmit beamforming solutions with a significantly reduced search space dimension.

### 5.3. The Number of Transmit Antennas

In this subsection, we examine the effects of the number of transmit antennas, *M*, on the average sum-rates of the proposed near-optimal transmit beamformers. From Figure 1 and Figure 2, it is numerically seen that regardless of *M*, the multiplexing gain of the proposed scheme is the same as the maximum multiplexing gain achieved by “Optimal scheme”. This implies that the multiplexing gain is invariant to *M* as far as M≥K. On the other hand, we can achieve the finite array gain (i.e., the power gain) with respect to increasing *M*, but an increasing rate of the array gain is reduced as *M* increases. When *M* is sufficiently large, the array gain is expected to be saturated to a certain level.

### 5.4. The Correlation Coefficient

In this subsection, we examine the effects of the correlation coefficient ρ on the average sum-rates of the proposed scheme. Recall that the design of our transmit beamformers is built upon the assumption that the correlation between two adjacent coding blocks is sufficiently high. Hence, it is easily expected that the proposed scheme shows comparable performance to “Optimal scheme” under highly correlated block fading environments, which is validated in Figure 3 and Figure 4. In addition to this argument, we observe that the proposed scheme is robust to the correlation of adjacent channel coding blocks. Thus, the period of updating optimal transmit beamformers can be further extended without substantial performance degradation.

### 5.5. Discussion

From the practical viewpoint, we can expect the throughput behavior under an imperfect CSI, where a mobile equipment includes a channel estimator for estimating all the channel coefficients in the Communication Processor (CP). Even though the channel estimator has a sophisticated signal processing, it is inevitable to have a channel estimation error, which results in effective noise enhancement. Here, the effective noise enhancement, i.e, noise variance increase, becomes more severe in the high SNR regime. Consequently, throughput performance degradation is inevitable especially in the high SNR regime. For overcoming the performance degradation, the channel estimation period needs to be decreased in order for the channel estimation error to be minimized.

## 6. Conclusions

In this paper, we have proposed a novel design of transmit beamformers that guarantees the near-optimal performance on the sum-rates in the multiuser MISO IC. The main idea behind our design methodology is to leverage side information obtained from the channel correlation between two adjacent coding blocks under our correlated block fading setting—more specifically, the received SINR for the previous coding block was used for designing beamformers. We provided a method for deriving closed-form solutions to effective beamforming in both low and high SNR regimes. The proposed method is based on a linear algebraic approach, which turned out to be more efficient than the optimal scheme. In addition, the proposed scheme finds the optimal transmit beamforming solution at each channel instance via exhaustive search, in the sense of reducing the average number of search space dimensions for designing transmit beamformers. Numerical results showed that the proposed scheme exhibit near-optimal sum-rates via computer simulations. It was also observed that the proposed scheme is robust to the correlation of adjacent channel coding blocks. Thus, it was confirmed that the period of updating optimal transmit beamformers can be extended without significant performance degradation while leading to a remarkable reduction in the computational complexity in comparison with other benchmark schemes. For future work, the extension of our work to multiple-input multiple-output (MIMO) systems could be an interesting issue under correlated fading IC environments. However, it is more challenging than the multi-user MISO IC because more sophisticated beamforming design is required for managing multi-user interference effectively, which has been partially considered in [27,28]. 

## Figures and Tables

**Table 1:** Search space dimension of the proposed beamformer and the conventional beamformers.

Search Space Dimension for Each Coding Block (K≥3)			
[1]	[5]	[8]	Proposed
2K(K−1)	K(K−1)	2K−2	0
